# Feasibility study of a novel mHealth clinical decision support application to enable community health workers to manage hypertension in rural Guatemala

**DOI:** 10.7189/jogh.16.04232

**Published:** 2026-07-31

**Authors:** Sean Duffy, Alejandro Chavez, Ian Stanley Arthur, Gabriella Lucia Ortiz, Alvaro Bermudez-Cañete, Pablo Nuñez-Perez, Elizabeth White, Valerie Aguilar, Juan Aguirre, Do Dang, Celina Sonia Perez Abaj, Yoselin Emelina Letona López, Rafael Tun, Taryn McGinn Valley

**Affiliations:** 1 University of Wisconsin School of Medicine and Public Health, Department of Family Medicine and Community Health, Madison, Wisconsin, USA; 2 University of California-San Francisco School of Medicine, San Francisco, California, USA; 3 University of Wisconsin-Madison, Madison, Wisconsin, USA; 4 Stanford University, Department of Bioengineering, Stanford, California, USA; 5 Brellium, Inc., New York City, New York, USA; 6 Medical College of Wisconsin, Department of Paediatrics, Milwaukee, Wisconsin, USA; 7 Northwestern University Feinberg School of Medicine, Department of Family and Community Medicine, Chicago, Illinois, USA; 8 NYU Langone Health, Division of Geriatric Medicine and Palliative Care, New York City, New York, USA; 9 Hospital Obras Sociales Monseñor Gregorio Schaffer, San Lucas Tolimán, Sololá, Guatemala

**Keywords:** hypertension, community health workers, mHealth, clinical decision support systems, global health, task shifting

## Abstract

**Background:**

Hypertension is a leading global cause of morbidity and mortality, especially in low- and middle-income countries, where low healthcare access limits diagnosis and treatment. Community health workers (CHWs) supported by mobile health (mHealth) clinical decision support (CDS) tools may help. This study evaluated the feasibility of CHW-led hypertension management using an mHealth CDS application in rural Guatemala.

**Methods:**

We conducted a single-group feasibility study in San Lucas Tolimán, Guatemala. Trained CHWs assisted by a CommCare-based CDS application provided direct patient care. Application algorithms were based on World Health Organization guidelines and assisted with medication titration, lifestyle counselling, and physician consultation. Adults (≥18 years) with diagnosed hypertension were followed monthly for six months. The primary feasibility outcome was prescribing agreement between CHWs and supervising physicians with application of antihypertensive recommendations (minimum acceptable agreement was 90%). Primary clinical outcomes were changes in systolic (SBP) and diastolic (DBP) blood pressure. Secondary outcomes included retention, patient satisfaction, and safety.

**Results:**

In total 32 participants were enrolled and 30 (93.8%) completed six-month follow-up, with 96.4% of possible visits completed. CHW-physician agreement with application recommendations was 98.9%. The median decrease in SBP was 7.5 mm Hg (95% confidence interval (CI) = 1.0, 12.0), and the mean decrease in DBP was 3.1 mm Hg (95% CI = 0.1, 6.1). The proportion of patients with controlled SBP (<140 mm Hg) increased from 66.7% to 76.7% (*P* = 0.505). A total of 11 application errors (5.0% of 217 visits) occurred, none of which resulted in adverse events. Only three patients experienced significant adverse events, none of which required hospitalisation. Patient satisfaction remained high.

**Conclusions:**

In this pilot, CHWs supported by an mHealth CDS application safely managed hypertension with high physician agreement, patient retention, and blood pressure improvement. These findings demonstrate the feasibility of CHW task-sharing hypertension management in low-resource settings and support evaluation of this approach in larger trials.

**Registration:**

ClinicalTrials.gov: NCT05479097

Hypertension is the most common chronic disease in adults and is a leading global cause of morbidity and mortality. Around a third of all adults, approximately 1.4 billion people, are estimated to have hypertension globally [1]. The prevalence of hypertension, along with the rate of hypertension-related deaths, is now higher in low- and middle-income countries (LMICs) than in high-income countries [1]. Many patients are able to effectively treat and manage their hypertension through lifestyle changes and affordable medications. However, successful management requires continuous monitoring and interaction with health services [2]. In LMICs, lack of access to skilled clinicians and medications contributes to a higher prevalence of uncontrolled hypertension and premature mortality [1]. Given the magnitude of hypertension as a public health problem, increasing access to effective treatment needs to be a global health priority [3].

Community health workers (CHWs) are frontline public health workers who can serve as a vital link between communities and health systems, connecting people to information, resources, and services [4]. Given LMICs’ lack of primary care infrastructure and skilled providers to treat hypertension, CHWs have been explored as a potential area of investment to increase capacity and reduce morbidity and premature mortality. Previous studies have shown that CHWs can effectively support hypertension management within health systems in LMICs [5]. However, CHWs’ involvement in hypertension treatment has primarily been limited to supportive and educational roles [6]. There is currently insufficient evidence on how CHWs can provide direct patient care, including whether mobile health (mHealth) clinical decision support (CDS) tools are feasible for CHWs to use under remote supervision, and whether these approaches could adequately support and manage patients. Several interventions utilising CHWs and other frontline health workers in direct patient care roles for hypertension management in LMIC have been described [7–11]. However, such interventions had important limitations that may inhibit more general uptake to fill gaps in hypertension care in LMICs, including requirement for physician approval of antihypertensive medication recommendations [7–9]; use of fixed-dose, single-pill antihypertensive regimens which may not be available in many areas [8]; lack of support for antihypertensive titration by front-line health workers [10]; and use of highly educated and trained health workers [11].

This study was conducted in San Lucas Tolimán (SLT), a municipality in the western highlands of Guatemala. Given the limited primary care infrastructure, SLT is an important test case for implementing chronic disease management programmes in LMICs. In SLT, healthcare is fragmented and often difficult to access [12]. Although Guatemala’s constitution enshrines a right to universal coverage, in practice, the public system is grossly underfunded with inconsistent access to primary care as well as medications, laboratory tests, and specialists [12–14]. Although there are private and non-governmental (NGO) options for medical care, many patients in SLT and surrounding areas reported difficulty paying for such services [12]. Along with the limitations of the healthcare system, the complex topography of SLT and deficient public infrastructure make it a useful setting for testing generalisability to other LMICs. SLT is found within a mountainous region with limited public transport, many unpaved roads, and inconsistent access to running water and electricity. Given the numerous barriers to logistics and transport, a successful programme in SLT could be applied to a variety of other settings.

In this study, conducted in rural areas of Guatemala with poor primary care infrastructure, we aimed to address the gap in research on direct hypertension management through CHWs and mHealth CDS tools. Community health workers have been shown to be effective in ancillary roles such as education, diagnosis, and monitoring, but there is limited evidence on whether CHWs can serve as the primary clinician with support from a CDS application. Improving task sharing between CHWs and local physicians in LMICs could help bolster primary care in settings with limited healthcare infrastructure. To strengthen this scholarship, we performed a pilot study to show the feasibility of this approach. By proving the feasibility of CHW-led hypertension management enabled by a mobile application, our study facilitates future testing of this approach in a larger trial.

## METHODS

### Study setting

In Guatemala, SLT is a rural municipality in the western highlands, located on the shores of Lake Atitlan. The municipality has a population of 35,000, including a town with 20,000 residents and another approximately 15,000 living in small surrounding communities. The majority of inhabitants in SLT are Indigenous Kaqchikel Maya. The most common sources of income are agricultural labour from coffee or banana plantations, or informal labour such as weaving. We conducted the study in the rural communities of SLT with monthly visits occurring in community spaces, churches, or classrooms. The leading Guatemalan organisation for this project was the San Lucas Mission (SLM), a non-profit organisation providing healthcare and other social services to the people of SLT and neighbouring municipalities. San Lucas Mission sponsors a CHW network serving the rural communities of SLT.

### Mobile application development

Over a 12-month period, we adapted our successful CDS application, designed to assist CHWs with diabetes management in rural Guatemala [15], to provide guidance for hypertension management and cardiovascular risk reduction. As with our diabetes application, this application was developed on the CommCare platform (Dimagi, Cambridge, Massachusetts, USA), which is widely used by frontline health workers in LMICs. Algorithms for titrating antihypertensives embedded in this application were based on the World Health Organization (WHO) HEARTS protocol for hypertension management [16] (Appendix S1 in the **Online Supplementary Document**).

We chose amlodipine, a calcium channel blocker; enalapril, an angiotensin-converting enzyme inhibitor (ACEI); and losartan, an angiotensin II receptor blocker (ARB), for our titration algorithms, based on international guidelines [16,17]. Due to lower cost and greater availability in Guatemala and in LMICs [1,18], we designated an ACEI (enalapril) as the first-line renin-angiotensin-aldosterone system inhibitor, with losartan (an ARB) used for patients with enalapril or other ACEI intolerance or who were taking an ARB at enrolment. The algorithms are capable of initiating and adjusting dosages for amlodipine or enalapril/losartan, used as monotherapy or combination. The application also provides guidance on statin and aspirin treatment for patients with a prior history of clinical atherosclerotic cardiovascular disease, and on statin treatment for primary prevention in high-risk patients, according to WHO and USA consensus guidelines [16,19]. The statin used for this study was atorvastatin.

The algorithms also utilise standard criteria to determine medication contraindications and discontinue relevant medications (or avoid initiating them) (Appendix S2 in **Online Supplementary Document**). The supervising physician can modify these contraindications and set maximum doses for use by the titration algorithm. In addition to medications managed by application protocols, the supervising physician can manually add antihypertensives, such as hydrochlorothiazide. While the application does not provide recommendations for titrating these additional medications, it recommends continuing them if blood pressure (BP) is within range and the patient reports no side effects. When these parameters are not met, the application prompts a real-time physician teleconsultation for medication guidance.

The application provides a rationale for its medication recommendations, allowing the user to independently verify the decision logic. The application also supports medication dispensing by calculating the number of pills needed to dispense for each medication based on the date of the next monthly visit and each patient’s remaining medication supply. For patients with low medication adherence based on pill count (when available) or self-report, the application prompts the CHW to reinforce adherence by encouraging patients to take medications at the same time each day, use a phone or watch alarm as a reminder, and keep the medications stored in a fixed place that is clearly visible.

In addition to medication titration algorithms, the application also provides guidance for lifestyle counselling, the identification of possible complications of hypertension – including hypertensive emergency, coronary artery disease, heart failure, and stroke – and remote consultation with a supervising physician as needed. Prior to the study, we conducted extensive quality-assurance testing on the application. To do so, we first fed dummy data into the application covering all possible scenarios to ensure that all recommendations provided by the app were accurate. Then, we observed CHWs using the application in simulated patient encounters to assess usability, fine-tune clarity of prompts and recommendations, and uncover unanticipated workflow problems and application errors.

### Community health worker training

To be eligible to participate in the study, CHWs had to have already completed the 24-month basic CHW training programme focused on care of common conditions and community health promotion. Eligible CHWs then completed a hypertension-specific curriculum consisting of five six-hour training sessions over a six-month period, focusing on standardised BP measurement, antihypertensive treatment, identification of potential complications or complex scenarios needing physician consultation, lifestyle guidance, and application use. Readiness to participate in the study was assessed by CHWs scoring 80% or higher on a written hypertension knowledge test and satisfactory performance on a practical examination, during which a trained observer used a standardised rubric to grade the learner’s use of the mobile application to assess and treat a standardised patient, as well as their BP measurement technique.

### Study design, sample, recruitment, and reporting

We conducted a single-group feasibility study. Target enrolment was 30 patients, which we found sufficient to assess the initial feasibility of our diabetes intervention [20] and falls within the recommended range for feasibility study sample sizes [21]. Inclusion criteria were: subjects aged ≥18 years with a physician-confirmed diagnosis of hypertension and BP≥140/90 or currently taking antihypertensives. Exclusion criteria included pregnancy and comorbid conditions leading to a life expectancy <1 year. We recruited patients from the rural communities of SLT, targeting individuals identified as having hypertension in our earlier hypertension screening study [22].

We used the CONSORT 2010 extension for pilot and feasibility trials checklist [23] to report study conduct and outcomes (Appendix S3 in the **Online Supplementary Document**).

### Study procedures

Patients met CHWs monthly, using the CDS application to guide each visit. At each visit, CHWs measured BP and weight, assessed medication adherence and tolerance, evaluated for possible complications of hypertension, provided lifestyle guidance, and dispensed medication. Height was also measured at the enrolment visit, and this measurement was used to calculate body mass index (BMI) in combination with weight (BMI = weight (kg)/height (m^2^)) at all time points. Community health workers measured BP using Omron M6 Comfort automatic upper arm BP monitors, which have been validated by the STRIDE BP Initiative [24]. The CHWs followed a standard protocol for measuring BP, which was adapted from International Society of Hypertension and WHO guidelines [17,25] (Appendix S4 in the **Online Supplementary Document**).

A nurse-level CHW coordinator provided direct supervision for the four to six CHWs attending to patients at each monthly check. Teleconsultation with a supervising physician was available as needed during study visits. Following each visit, application data was uploaded to the secure CommCare server, and a supervising physician reviewed the current care plans and adjusted them as needed in coordination with CHWs. The total duration of study participation was six months.

At the enrolment and six-month follow-up visits, a fasting blood draw was completed by venipuncture to measure serum creatinine, potassium, lipid profile, glucose, and alanine aminotransferase (ALT). Laboratory processing, entry of results, and physician review of results would occur within 72 hours of blood draw. At these visits, patients also completed validated Spanish versions of the Hypertension Self-Care Activity Level Effects (H-SCALE) [26], the Patient Assessment of Care for Chronic Conditions (PACIC) [27], and a single-item measure of global patient satisfaction [28].

To assess patient perspectives, a qualitatively trained researcher and a CHW conducted interviews with each patient after the six-month study visit. The team used a semi-structured interview guide (which was collaboratively developed with the lead researcher, a qualitatively trained team member, and the CHW team) to allow for some standardisation while also enabling patients to bring up any issues or suggestions (Appendix S5 in the **Online Supplementary Document**). All patients were visited at home to request participation in interviews; if they were not present at home during the first visit, they were visited again. Patients were not required to participate in interviews to continue receiving study treatment. Informed consent was obtained for recording and participation in the approximately 20-minute interview as part of the larger study. Interviews were conducted in Spanish, Kaqchikel, or a combination, depending on patient preference. Interviews were recorded, and the qualitative researcher took synchronous notes.

### Outcome measures

#### Primary outcomes

The primary feasibility outcome was clinician agreement with the monthly antihypertensive prescription recommended by the CDS application. We assessed this as the proportion of visits for which both the CHW conducting the visit and the physician reviewing the data after the visit agreed with the antihypertensive recommendations provided by the application. We used this as our primary feasibility metric because we felt this approach would reflect the overall reliability of the application’s CDS. A low level of agreement would indicate the need for frequent consultation between CHWs and physicians during visits and between visits for medication changes, increasing workload and reducing the feasibility of the intervention. We set a target threshold of at least 90% agreement. While there is no widely agreed-upon standard for this measure in such programmes, studies of successful CDS applications used by non-physician health workers have found analogous agreement proportions exceeding 90% [8,15]. We assessed agreement over the final three months of the trial, as we anticipated that agreement would improve throughout the trial with iterative application improvements. As a *post hoc* sensitivity analysis, we assessed the impact of excluding visits in which a real-time consultation with the supervising physician led to a change in medication recommendations on the clinician agreement metric.

Primary clinical outcomes included changes in systolic BP (SBP), diastolic BP (DBP), and hypertension control status (defined as SBP<140 mm Hg). We used SBP<140 mm Hg as a measure of BP control to mirror other studies evaluating task sharing with CHWs for hypertension care [8–10]. Epidemiological studies have also demonstrated that SBP has a greater influence on cardiovascular outcomes than DBP, particularly for patients aged >50 years [29,30]. As *post hoc* sensitivity analyses, we also assessed the change in hypertension control status determined by combined SBP and DBP (defined as BP<140/90 mm Hg) and stratified the change in BP by baseline control status. Unless otherwise noted, all clinical outcomes were assessed at six months.

#### Secondary outcomes

Secondary feasibility outcomes included subject retention, visit completion, the frequency and type of real-time physician consults, and patient global satisfaction and PACIC scores. The feasibility assessment also included usability surveys and feedback from CHWs who participated in the study, as described elsewhere [31].

Secondary clinical outcomes included changes in weight, serum creatinine (and calculated estimated glomerular filtration rate (eGFR), lipid profile, glucose, hypertension self-care activities (H-SCALE scores), and medication adherence. When available, the pill count adherence ratio (PCAR) [32] was used to determine adherence, with self-reported adherence used only when PCAR was unavailable. PCAR represents the ratio of pills actually consumed to the number of pills that should have been consumed in a given time period with perfect adherence, calculated by the formula: pills dispensed (n) – pills left (n)/pills that should have been consumed (n). To ensure accuracy of this measure, PCAR was only considered valid for a given check if subjects brought (or received if the enrolment visit) their pill box to the previous monthly check, brought their pill box to the current check, and there were no medication changes between checks. Acceptable adherence was defined as PCAR≥75% or, for self-report, reporting taking medications as prescribed on at least five of seven days/week. As the pill count was unavailable at baseline because patients had not yet been dispensed medication, we used self-report to assess baseline adherence. We compared baseline adherence with adherence at six months, and adherence at month one with month six for cases in which PCAR was available at both time points.

#### Safety outcomes

Safety outcomes included the incidence of software errors or malfunctions resulting in incorrect medication recommendations provided by the CDS application, user errors resulting in incorrect medication recommendations, and the incidence of complications of hypertension or antihypertensive treatment, including a significant increase in serum creatinine (≥1.5 × baseline), acute coronary syndrome, cerebrovascular accident, decompensated heart failure, hyperkalaemia, and syncope. Particular errors were identified by CHWs, the supervising nurse, or the supervising physician during the review of the monthly visits.

### Data analysis

As this study focused on determining the feasibility of CHW-led hypertension management, power analyses were not performed. We used *R*, version 4.5.2 (The R Foundation for Statistical Computing, Vienna, Austria) for all statistical analyses.

For continuous outcomes, we used the Shapiro-Wilk test to determine normality. We summarised normally distributed variables using means and standard deviations, and non-normally distributed variables using medians and interquartile ranges. We assessed changes in paired measures from baseline to six months *vs.* a null hypothesis of no change using paired *t* tests (for normally distributed variables) or Wilcoxon signed-rank tests (for non-normally distributed variables). For dichotomous outcomes, we calculated raw proportions for baseline and follow-up periods. We assessed the association between the outcome and the two periods *vs.* a null hypothesis of no association using McNemar’s test. The statistical significance threshold for all analyses was ɑ = 5%. All analyses other than those noted as *post hoc* were prespecified. As this was a feasibility (exploratory) study aimed at informing design and plans for a larger controlled trial, we did not adjust *P*-values for multiple comparisons [33].

### Qualitative analysis

The same qualitatively trained researcher who conducted interviews transcribed interviews in Spanish and Kaqchikel. An inductive-deductive coding method was used to both attend to *a priori* themes covered in the questions and allow *de novo* themes to emerge from the transcribed and audio data. The researcher who conducted the interviews completed transcription, analysis, and coding following ethnographic methods, and engaged in contemporaneous memoing to reflect on emerging and existing codes. Then, this researcher had coding meetings with a small group of other team researchers who had conducted qualitative research and participated in the study design and completion. This combination of coding, reflexivity, memoing, and group discussion led to common themes. These themes were then collectively assigned to coded areas of the transcripts and finally discussed with the community health workers and broader research teams for member checking and triangulation.

## RESULTS

### Participants

A total of 32 subjects participated in the study (**Table 1**). The study population was heavily skewed towards female participants (84.0%), and a large majority entered already taking antihypertensives (97.0%); most began with controlled SBP (66.0%). Enrolment occurred in February 2023, and study interventions continued through August 2023.

**Table 1:** Baseline characteristics of study participants*

Characteristics	n = 32
Age, Mdn (IQR)†	59 (51, 68)
Sex	
*Female*	27 (84)
*Male*	5 (16)
Diabetes	10 (31)
BMI, Mdn (IQR)	28.0 (27.1, 33.0)
SBP, Mdn (IQR)	132 (125, 144)
DBP, Mdn (IQR)	79 (74, 90)
Taking antihypertensives	31 (97)
SBP<140 mm Hg	21 (66)

BMI – body mass indexDBP – diastolic blood pressure, IQR – interquartile range, Mdn – median, SBP – systolic blood pressure

*Presented as n (%) unless specified otherwise.

†Age range of participants was 29–82 years.

### Primary outcomes

#### Agreement with the application of antihypertensive recommendations

CHW and physician agreement with antihypertensive medication recommendations was 98.9% (92 of 93 visits) over the last three months of the trial, exceeding the prespecified feasibility threshold of 90%. When the six visits during which antihypertensive medication therapy was altered by real-time physician consultation were excluded, agreement was 98.8% (86 of 87 visits). Agreement throughout the trial period was 96.7% (210 of 217 visits), which was also higher than the threshold. When the 11 visits during which antihypertensive medication therapy was altered by real-time physician consultation were excluded, agreement was 96.6% (199 of 206 visits). Of note, these agreement figures reflect physician agreement only; there were no instances in which CHWs disagreed with the treatment recommendations provided by the application.

#### Blood pressure

**Figure 1:**
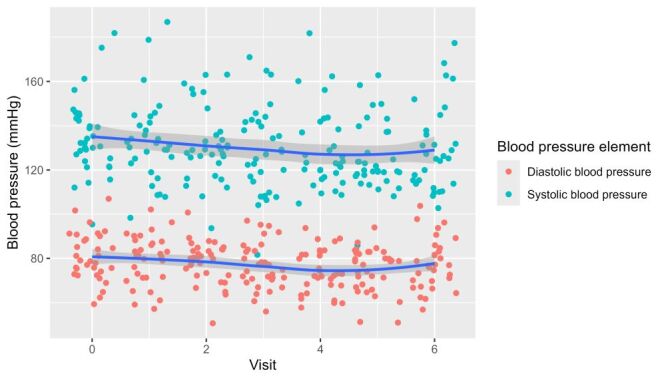
Blood pressure trends during pilot study

Median decrease in SBP was 7.5 mm Hg (n = 30, 95% confidence interval (CI) = 1.0, 12.0) from enrolment to six months. Mean decrease in DBP for this time period was 3.1 mm Hg (n = 30, 95% CI = 0.1, 6.1). Decreases in BP were greater in subjects with uncontrolled BP at baseline (**Table 2**). There was a general downward trend for SBP and DBP (**Figure 1**).

**Table 2:** Change in blood pressure stratified by baseline control

Items	Baseline*	6 Months*	Difference (95% CI)†	*P*-value†
SBP in mmHg, Mdn (IQR)				
*All (n = 30)*	132.0 (125.5, 142.8)	125.5 (114.2, 137.5)	–7.5 (–12.0, –1.0)	0.032
*Controlled at baseline (<140 mm Hg, n = 20)*	129.0 (121.8, 132.0)	122.0 (113.5, 133.0)	–4.0 (–9.0, 5.5)	0.29
*Uncontrolled at baseline (≥140 mm Hg, n = 10)*	146.0 (143.5, 153.8)	130.0 (125.2, 157.8)	–15.5 (–25.0, 1.0)	0.052
DBP in mmHg, x̄ (SD)				
*All (n = 30)*	80.8 (11.3)	77.7 (11.6)	–3.1 (–6.1, –0.1)	0.045
*Controlled baseline (<90 mm Hg, n = 22)*	75.6 (7.7)	72.8 (8.0)	–2.9 (–6.6, 0.9)	0.13
*Uncontrolled baseline (≥90 mm Hg, n = 8)*	95.0 (6.3)	91.4 (8.5)	–3.6 (–9.7, 2.4)	0.20

CI – confidence interval, DBP – diastolic blood pressure, IQR – interquartile range, Mdn – median, SBP –systolic blood pressure, SD – standard deviation, x̄ – mean

†Wilcoxon signed rank test with continuity correction (median difference reported); Paired *t* test (mean difference reported).

The percentage of patients with SBP controlled (<140 mm Hg) increased from 66.7% at baseline to 76.7% at six months (n = 30, McNemar’s χ^2^ = 0.444, *P* = 0.505) (**Table 3**). The percentage of patients with SBP and DBP controlled (<140/90 mm Hg) increased from 60.0% at baseline to 70.0% at six months (n = 30, McNemar’s Chi-squared = 0.444, *P* = 0.505) (Table S1 in the **Online Supplementary Document**).

**Table 3:** Blood pressure control status

Baseline control	Control at 6 months, n (%)		
	**No**	**Yes**	**Total**
No	4 (13.3)	6 (20.0)	10 (33.3)
Yes	3 (10.0)	17 (56.7)	20 (66.7)
Total	7 (23.3)	23 (76.7)	30 (100.0)

### Secondary outcomes

#### Subject retention and visit completion

Of the 32 subjects enrolled, 30 (93.8%) remained in the study through the six-month follow-up visit. One subject withdrew because of relocation to another city, and another withdrew due to side effects from antihypertensive medications. Subjects completed 96.4% of possible follow-up visits.

#### Real-time physician consultations

Consultation with a supervising physician occurred during 12.0% (26 of 217) visits. The most common reason for consultation (11 consults, 42.3% of all consults) was a medication scenario that fell outside of the antihypertensive titration protocols used by the application (Table S2 in the **Online Supplementary Document**).

#### Patient satisfaction

Subject ratings on the PACIC survey items were high at baseline, with a median score of five (indicating that the desired healthcare activities were performed ‘almost always’ when they sought hypertension care) for all items. Despite this, there were statistically significant improvements for six of the 11 survey items at six months, as well as in the average of all included survey items (Table S3 in the **Online Supplementary Document**).

There was no statistically significant change in global satisfaction with hypertension care from baseline to six months, with subjects reporting high satisfaction (median score of four, ‘satisfied,’ 95% CI = –1.5, 1.0, *P* = 0.71) at both time points.

#### Hypertension self-care activities

There were no statistically significant changes in H-SCALE sub-scores from baseline to six months (Table S4 in the **Online Supplementary Document**). Of note, the alcohol sub-score was not included in this analysis because all patients had a score of zero (no reported alcohol use) at baseline and six months.

#### Laboratory and anthropometric outcomes

There was a small but statistically significant increase in creatinine (and associated reduction in eGFR) at six months compared to baseline. There were also statistically significant reductions in triglycerides, glucose, and ALT. There were no statistically significant changes in BMI or weight from baseline to six months (Tables S5 and S6 in the **Online Supplementary Document**).

#### Medication adherence

All participants were 100.0% adherent by self-report at baseline, compared to 96% by either adherence measure at six months (n = 28, *P* = 1). Further, 93.0% of participants were adherent to PCAR at one month, which decreased to 79.0% at six months (n = 14, *P* = 0.617) (Tables S7 and S8 in the **Online Supplementary Document**).

### Safety outcomes

There were 11 application errors resulting in incorrect medication recommendations (5% of 217 total visits). All of these occurred in the first three months of the study and were promptly corrected through application updates. Moreover, eight of these errors represented the same application malfunction following the enrolment visit, which resulted in the failure of the application to recommend dispensing of statin medication when indicated at the first monthly follow-up visit. The issue was detected on routine pre-check testing by the study team and was manually corrected during the study visit. Another error occurred due to the incorrect updating of the prescribed dose of an antihypertensive at the end of a prior visit. This led to a discrepancy between what the patient reported taking at the following visit and what the application recorded as the correct dose, triggering a physician consultation during the visit, which led to the correct medication dose being dispensed. Moreover, two errors occurred because medication side effects reported the previous month were improperly carried forward to the following month, resulting in the medications being inappropriately decreased or changed to a different medication. These errors were discovered upon routine review of monthly visits by the supervising physician, were remedied by a medication change in one case within several days of the check and did not result in any adverse effects on subjects. There were three cases (1.2% of 217 total visits) in which CHW data-entry errors (rather than application malfunctions) resulted in incorrect medication administration, detected during physician review of monthly check data and remedied within several days of the check.

Regarding medication adverse events, one subject experienced a ≥50.0% increase in creatinine from baseline to six months, though creatinine remained within normal limits. Another subject had an episode of syncope related to antihypertensive medications. Regarding complications of hypertension, one subject experienced symptoms (dyspnoea on exertion and lower extremity oedema) suspected to be related to decompensated heart failure. None of these medication adverse events or hypertension complications required hospitalisation.

### Qualitative results

Of the 32 patients, 25 were at home during home visits and were interested in participating in an interview. These brief interviews lasted from three to 31 minutes, averaging eight minutes. Overall, patients reported feeling satisfied with and appreciative of the programme. In particular, patients appreciated that the care was free, included medicines, and took place close to their homes. Before the pilot programme, patients either paid out of pocket for medication or did not take it. Many took medication only when they could afford it, leading to spotty use, and they often went without.

In a response representative of most interviewees, one patient commented:

*This program is helpful because we get medicine. Buying medicine ourselves is expensive, not just buying it but even getting to town to buy it. If you don’t have the money, it’s impossible.* – Interview patient

One man commented that the programme seems to ‘especially help us who are really poor’ because it provides access to medications. She shared that she feels ‘much more comfortable now… thanks to God that this program is here helping us’.

Before the programme, one patient explained:

*I used to try to get them at the Government Health Centre, but sometimes I would have to go to a pharmacy instead, and whenever they would run out, I’d have to find a way to pay for them.* – Interview patient

Several patients reported taking no medicine before the programme, despite knowing about their hypertension diagnoses, because of prohibitive cost and transport.

We also asked patients what they would change or improve about the programme. A few patients requested treatment for non-hypertension concerns, *e.g.* allergies and headaches. Some, including a midwife whose access to supplies had been cut off when a government programme stopped working with traditional birth attendants, asked the study team for birth work tools, like gloves and wound care supplies. One patient, whose main hypertension symptom prior to enrolling in the study had been headaches, shared ‘my head hurts now, but nothing like before…but I think this headache now comes from not having enough vitamins.’ She described having had 12 children, and feeling that ‘all of my body’s vitamins have been used up’ by her many childbirths. She saw vitamins as a core part of healthcare and, like many interviewees, expressed a wish that the programme also provided them.

Overall, most patient interviewees said there was nothing they would change about the hypertension programme. More than one patient responded that they did not have the ‘right’ to suggest any changes; several said, ‘I don’t know what to say.’ One patient said:

*I can’t put myself in the position of saying anything bad. If I were to say anything was wrong with the program, I’d be lying. It’s a strong program and I feel good.* – Interview patient

In Guatemala, it is quite rare for patients to be asked for feedback about their biomedical care.

## DISCUSSION

In this pilot study of CHW-led hypertension management enabled by an mHealth application providing CDS, we found that this approach is feasible and shows initial indications of clinical safety and efficacy. CHWs were able to make reliable treatment decisions regarding antihypertensive therapy using the application, as the physician reviewing patient data agreed with these decisions greater than 95% of the time, higher than the prespecified feasibility target of 90% agreement. While the primary objective of this study was to demonstrate intervention feasibility, subjects also showed statistically and clinically significant reductions in BP (7.5 mm Hg and 3.1 mm Hg reductions in SBP and DBP, respectively), despite the fact that all but one subject (97%) entered the study already taking antihypertensives. There were no severe treatment-related adverse events, and subjects reported generally high satisfaction with CHW-led care, while we also found improved scores on several measures of the PACIC survey. Taken together, these findings suggest that using mHealth to support task sharing with non-physician health workers for hypertension care is a viable strategy to improve treatment access and outcomes in low-resource settings.

The primary feasibility measure for this study was the agreement of CHWs and supervising physicians on the antihypertensive recommendations made by the mHealth application. This metric reflects the ability of CHWs working within this system to function autonomously and effectively at the point of care – high agreement with application recommendations translates to less need to consult with a physician in real time during patient visits, fewer medication changes that need to be coordinated after visits, and improved patient safety due to fewer erroneous or clinically unsound medication prescribing decisions. Agreement over the course of this study was 96.7%, exceeding the prespecified feasibility threshold of 90%. This rate increased to 98.9% over the final three months of the trial, reflecting continuous improvements to the application based on end-user feedback. Notably, the agreement observed in this study is higher than that seen in our earlier study of CHW-led diabetes management (91%) [15] and in the Heart Outcomes Prevention and Evaluation (HOPE) 4 study (93%), which evaluated a collaborative care model between non-physician health workers and physicians utilising a CDS mHealth application [8].

Secondary outcomes were also supportive of intervention feasibility. Subject retention through six months was 93.8%, and subjects completed 96.4% of possible study visits. This is greater than the median retention of approximately 90% reported across a wide variety of clinical trials, as well as the commonly targeted retention rates of 85–90% [34–36]. Patient satisfaction measures were high at baseline. We theorise baseline scores in this medically underserved population may have been inflated by increased access to care in the months prior to the start of this pilot study; all subjects were recruited from our earlier study of hypertension diagnosis [22] and, because the start of the pilot treatment study was delayed, had been provided with prior, bridging antihypertensive medications managed by study physicians. Nevertheless, there were significant improvements in the majority of PACIC survey items, as well as in the total PACIC score. The overall rate of application errors leading to incorrect medication recommendations was low, affecting 5% of visits. Though this rate was higher than the <1% error rate observed in our earlier study of a similar diabetes application [15], all of these errors occurred within the first three months of the study, and more than two-thirds represented the same error related to statin initiation across multiple patients. This suggests high reliability of the application after early errors were identified and fixed.

The statistically and clinically significant improvements in BP observed at six months in this study, while not a primary feasibility outcome and subject to the limitations of this pilot study, compare favourably with those reported in other studies of task-sharing interventions for hypertension care in low-resource settings. A 2019 systematic review and meta-analysis of task sharing with non-physician health workers for BP management in LMIC reported mean reductions of 4.85 mm Hg in SBP and 2.92 mm Hg in DBP across all interventions, a smaller magnitude of improvement than we observed [37]. Further, BP reductions were more modest in the subset of studies involving CHWs – 3.67 mm Hg for SBP and 2.29 mm Hg for DBP [37].

While other trials have used similar care models, our study innovated on existing research. The HOPE-4 trial employed a programme design very similar to our intervention, utilising CHWs equipped with mHealth CDS applications to enable task sharing with primary care physicians in Colombia and Malaysia [8]. This trial found a greater reduction in mean SBP (20.1 mm Hg) and DBP at six months in the treatment group (6.1 mm Hg) than in our intervention. However, baseline BP was significantly higher in the HOPE-4 study cohort (SBP = 152.1 mm Hg and DBP = 84.7 mm Hg) than in our study (median SBP was 132 mm Hg and median DBP was 79 mm Hg), and therefore, average BP at six months was similar for the two studies – 132/78.6 mm Hg for HOPE-4 *vs.* 125.5/75.9 mm Hg for this study.

Other key differences between HOPE-4 and this study include CHWs’ autonomy and the complexity of CDS delivered *via* mHealth applications. In HOPE-4, physicians approved all medication changes prior to CHWs carrying them out, a two-step process for medication changes, likely increasing CHW and physician workload and potentially leading to gaps or delays in care. In contrast, supported by the CDS application, CHWs in our study made most medication changes at the point of care using standardised protocols, with retrospective physician review and approval. Additionally, while the HOPE-4 protocols used fixed-dose, single-pill antihypertensive combinations, the advanced decision support provided by the application in our study allowed for initiation and titration of three different antihypertensives, improving personalisation of antihypertensive therapy and generalisability to other LMIC settings, where availability and affordability of single-pill combination medications are currently suboptimal [38,39].

Another trial similar in concept to our study is the China Rural Hypertension Control Project (CRHCP), a cluster RCT randomising villages to hypertension care delivered by village doctors, a type of CHW operating in rural areas within the Chinese healthcare system, *vs.* usual care [11]. Our study also innovated compared to this useful study. In the CRHCP, village doctors were trained on standardised protocols for antihypertensive initiation and titration and were supported and supervised by primary care physicians. Subjects in the intervention group experienced impressive reductions in SBP and DBP at 18 months (26.3 mm Hg and 14.6 mm Hg, respectively). As in the HOPE-4 study, baseline SBP (157.0 mm Hg) and DBP (88.1 mm Hg) were significantly higher than in our pilot study, likely accounting for the greater reductions and resulting in similar average BPs at study endpoint in both trials (130.7/73.5 mm Hg in CRHCP *vs.* 125.5/75.9 mm Hg for our study). There are also significant differences in the educational background of the village doctors utilised in the CRHCP and the CHWs who carried out the intervention in our study. Nearly 95% of the village doctors had completed at least high school or a vocational medical school, and more than 60% had completed a junior medical college or above. In comparison, only one of the six CHWs who participated in this pilot study had completed any formal education beyond the equivalent of middle school. The use of a pilot mHealth clinical decision application was crucial to enabling CHWs with less formal education to provide hypertension care, an innovation not present in the CRHCP study. While acknowledging the pilot nature of this study, we believe that the mHealth strategy we employed increases the potential for wider application of our model in LMIC settings following further testing. The relative affordability of CHWs compared with health workers with professional degrees is a key strength of this approach, given funding limitations and the dearth of physicians throughout Guatemala and other LMICs [40]. We believe that through the CDS application, CHWs could help broaden primary care support for hypertension, helping bring systems closer to universal coverage [40].

Our pilot study found no statistically significant improvements in hypertension self-care activities, as measured by the H-SCALE questionnaire. Additionally, while medication adherence at the beginning of the study was exceptionally high (100% by self-report at enrolment and 93% by PCAR at one month) and left little room for improvement, our pilot data suggest a non-statistically significant decrease in medication adherence over the course of the study. CHW training in preparation for this study emphasised lifestyle management of hypertension, and the mobile application supporting the CHWs provided prompts to deliver patient education on lifestyle changes and to provide medication adherence counselling. However, these findings suggest that more structured and intensive educational interventions may be required to bring about significant improvements in patient hypertension self-care and medication adherence. We find it likely that education-based interventions in larger studies, including our forthcoming trial, will succeed or fail more based on local context and collaboration than anything intrinsic to the CDS tool.

In our small pilot sample, we observed a small but statistically significant increase in creatinine (and a corresponding reduction in eGFR) over the course of the study (0.1 mg/dL, or 10.7%). Rather than representing a true worsening of renal function, we feel that this most likely represents the impact of initiation or escalation of ACEI or ARB therapy during the study, which typically causes a creatinine increase of 10–30%, despite long-term reno-protective effects [41,42]. We observed a syncope rate of one event per 16 patient-years. In comparison, in the control arm of the ‘SPRINT’ trial, which had the same SBP goal of <140 mm Hg as this study, the rate of syncopal events was one per 206 patient-years [43]. Given the small size of our study, we suspect that this difference in rates is primarily due to random chance rather than a significantly increased risk of syncope. A larger trial will be necessary to determine the true risk of syncope and other adverse effects of antihypertensive treatment.

Overall, our qualitative data supported that patients found this small pilot intervention both feasible and acceptable. Indeed, interviewed patient participants had few negative or constructive comments. However, given the nature of power differentials and healthcare systems in rural Guatemala, it is difficult to conclude that patients had no constructive feedback. We made the pragmatic and ethnographic decision to have the same study team members, who knew patients well, conduct interviews, but this certainly led to social desirability bias, skewing our results towards positive feedback. We do not necessarily believe, though, based on previous experience in the community and region, that having an interviewer not known to patients would have led to more honest feedback; instead, we suggest that directly asking patients what they would change about programmes is likely not the most useful way to elicit programme areas for improvement in communities with such explicit power differentials with NGO and foreign healthcare projects. Therefore, while the qualitative data increased our comfort with this pilot intervention, we are revising our qualitative methods for future programme evaluation.

This pilot study, while only preliminary to prove feasibility, was made possible by specific factors of our context and local partners, which may be helpful to list for other groups interested in initiating or expanding similar projects. We trained a larger group of CHWs but believe minimum staffing for a programme caring for up to 150 patients would include: 1.5 CHW full time equivalents (FTEs) with having at least four part-time CHWs optimal; one project coordinator (0.25 FTE) with capacity to train CHWs on application and patient care skills; one local physician (0.10 FTE) with remote oversight and supervision capacity, one local laboratory technician (0.20 FTE) with access to a laboratory; mobile device access for all involved personnel; internet connectivity (though asynchronous connection to the internet would be sufficient); and budget to buy aforementioned medications and supplies, and pay salaries for the above listed health workers. As we have already detailed, local collaborators and community-wide buy-in will be essential for any similar project to succeed.

An inherent limitation of this study was the small sample size, which limits inferences about intervention effectiveness in improving clinical outcomes. Additionally, we did not adjust statistical analyses for multiple testing, which may have increased the risk of false positives. Nevertheless, we feel that this sample size and our analysis were sufficient to demonstrate the feasibility and effectiveness of the intervention, which is currently being assessed in a much larger, controlled trial [44].

The fact that most participants were receiving regular hypertension care and taking medications at baseline also limited our ability to assess the effects of the intervention on newly diagnosed patients or those without regular access to care. However, we observed clinically significant improvements in BP and other health measures, which supports the feasibility of the intervention. We acknowledge that many of the successes of this programme had to do with our longstanding collaborations in the study communities; our preliminary data suggest that the CDS allowed us to further leverage an existing health programme in rural Guatemala to expand hypertension care.

## CONCLUSIONS

In this study of mHealth-enabled hypertension care provided by CHWs, we found that this innovative care model was feasible, safe, and improved BP and other clinical outcomes. We are currently assessing the efficacy of this approach in a cluster randomised, non-inferiority clinical trial comparing direct CHW care with care provided by a physician. This larger trial in particular has implications for task-sharing in LMIC for chronic disease care, and we hope our pilot model is helpful to others addressing hypertension in similar settings. In addition to our own cluster trial, future directions for other research projects could be innovated based on the findings from this study. Indeed, the findings from this feasibility study informed refinements that we made to the CHW care model prior to the start of our larger trial, including: the addition of diabetes management functionality to the application used by CHWs, given the high coincidence of diabetes and hypertension in the study cohort; and the inclusion of more structured patient education provided by the CHWs, as well as educational text messaging to patients. We are hopeful that our larger trial, facilitated by this pilot data, will demonstrate the non-inferiority of this task-sharing approach compared to traditional, physician-centric care for hypertension and thus provide another powerful tool for improving patient outcomes in low-resource settings.

## Additional material


Online Supplementary Document

## Data Availability

**Data availability:** The data sets generated and/or analysed during the current study are not publicly available, as sharing outside the research team was not part of the research protocols approved by the ethical oversight committees for this project, but may be made available upon reasonable request to the corresponding author with IRB permission.
