# Supplementary material for: Feasibility study of a novel mHealth clinical decision support application to enable community health workers to manage hypertension in rural Guatemala
Source: J Glob Health. 2026 Jul 31;16:04232. doi: 10.7189/jogh.16.04232 (PMC13424920; doi:10.7189/jogh.16.04232)
Supplement: Online Supplementary Document [file jogh-16-04232-s001.pdf]

Supplement to: **Duffy S, Chavez A, Arthur IS, Ortiz GL, Bermudez-Cañete A, Nuñez-Perez P, White E, Aguilar V, Aguirre J, Dang D, Perez-Abaj CS, Letona-López YE, Tun Rafael, Valley TM. Feasibility study of a novel mHealth clinical decision support application to enable community health workers to manage hypertension in rural Guatemala. J Glob Health. 2026;16:04232.**

Appendix S1 - Medication titration algorithms

Calcium channel blocker (CCB) titration algorithm

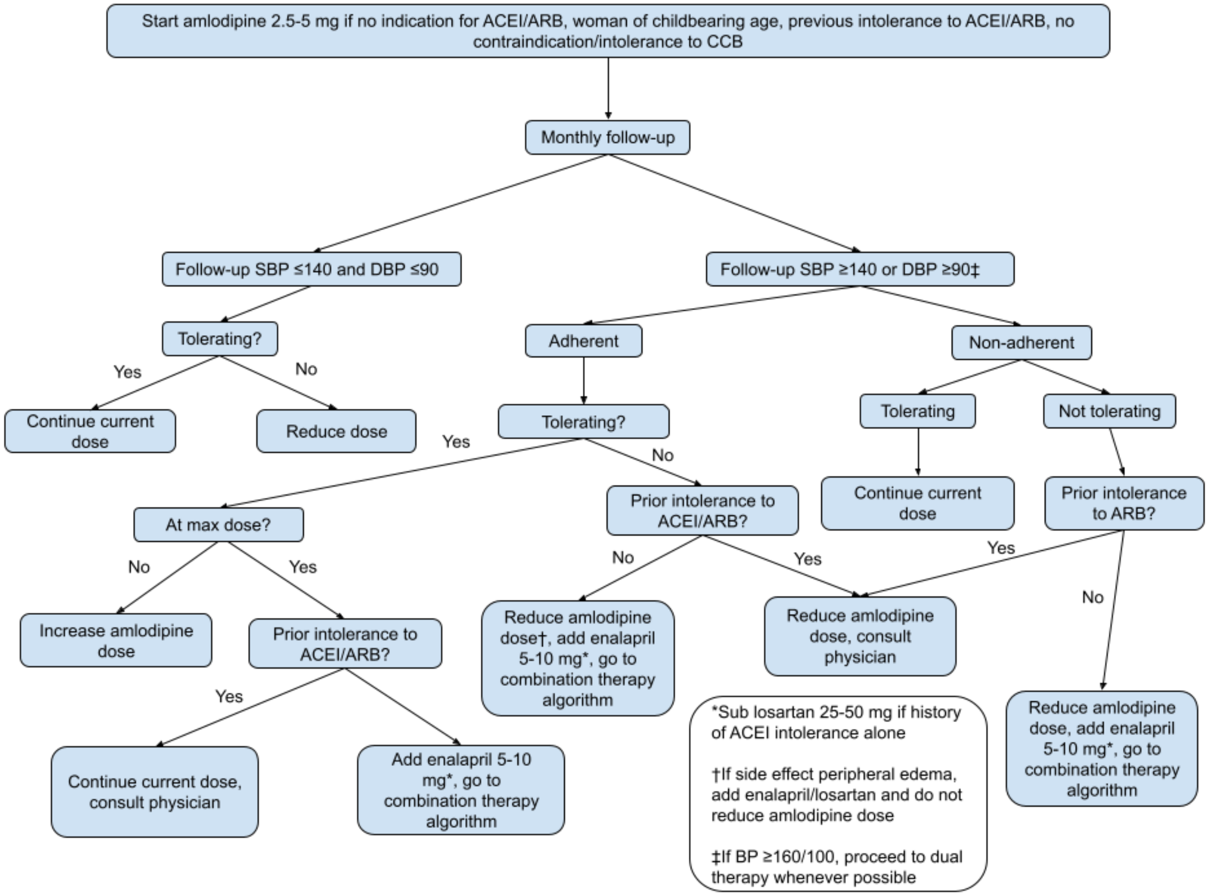

Angiotensin-converting enzyme inhibitor (ACEI) titration algorithm

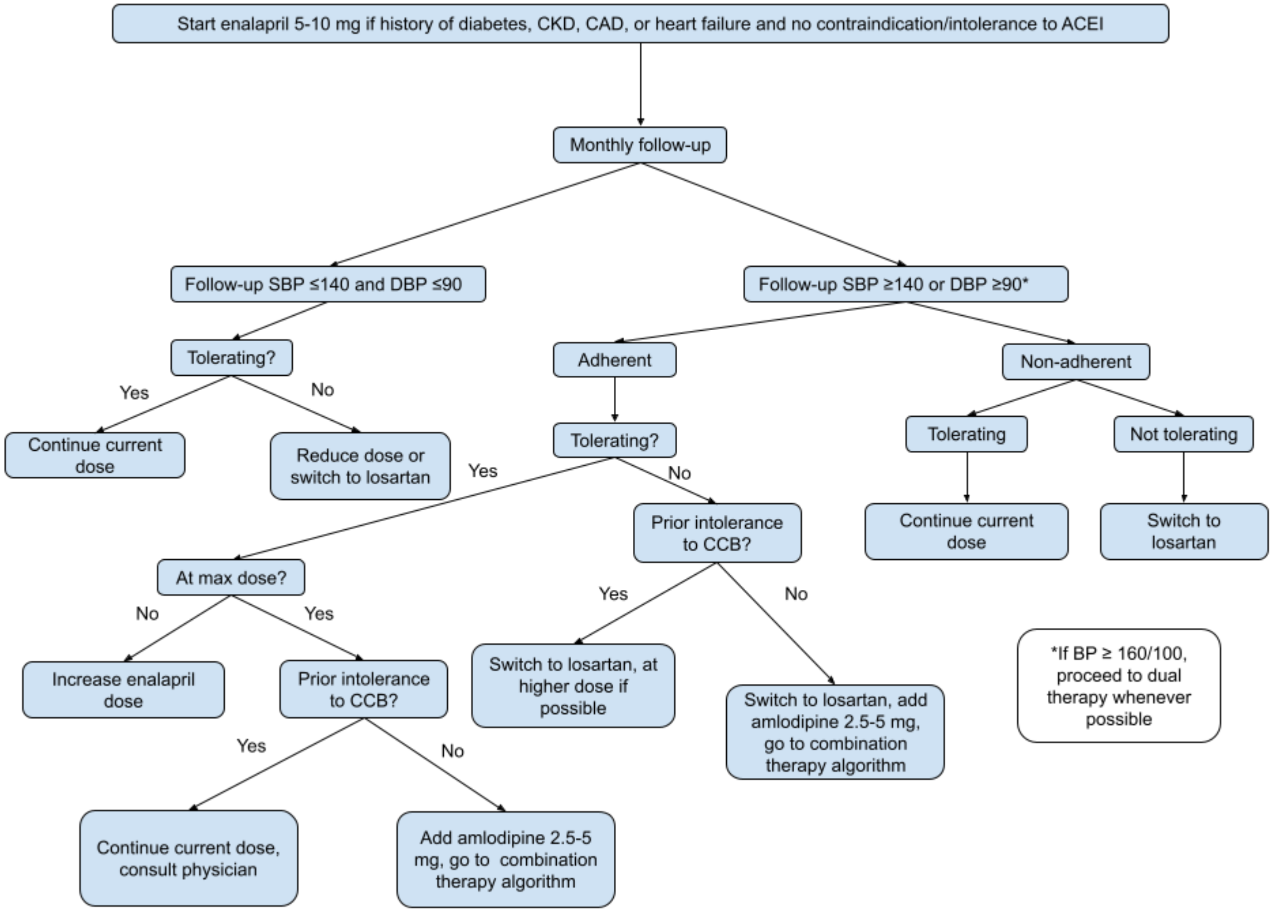

Start enalapril 5-10 mg if history of diabetes, CKD, CAD, or heart failure and no contraindication/intolerance to ACEI
Monthly follow-up
Follow-up SBP ≤140 and DBP ≤90
Follow-up SBP ≥140 or DBP ≥90*
Tolerating?
Yes
No
Continue current dose
Reduce dose or switch to losartan
Adherent
Non-adherent
Tolerating?
Yes
No
Tolerating
Not tolerating
Continue current dose
Switch to losartan
Prior intolerance to CCB?
At max dose?
No
Yes
Increase enalapril dose
Prior intolerance to CCB?
Yes
No
Continue current dose, consult physician
Add amlodipine 2.5-5 mg, go to combination therapy algorithm
Yes
No
Switch to losartan, at higher dose if possible
Switch to losartan, add amlodipine 2.5-5 mg, go to combination therapy algorithm
*If BP ≥ 160/100, proceed to dual therapy whenever possible

Angiotensin receptor blocker (ARB) titration algorithm

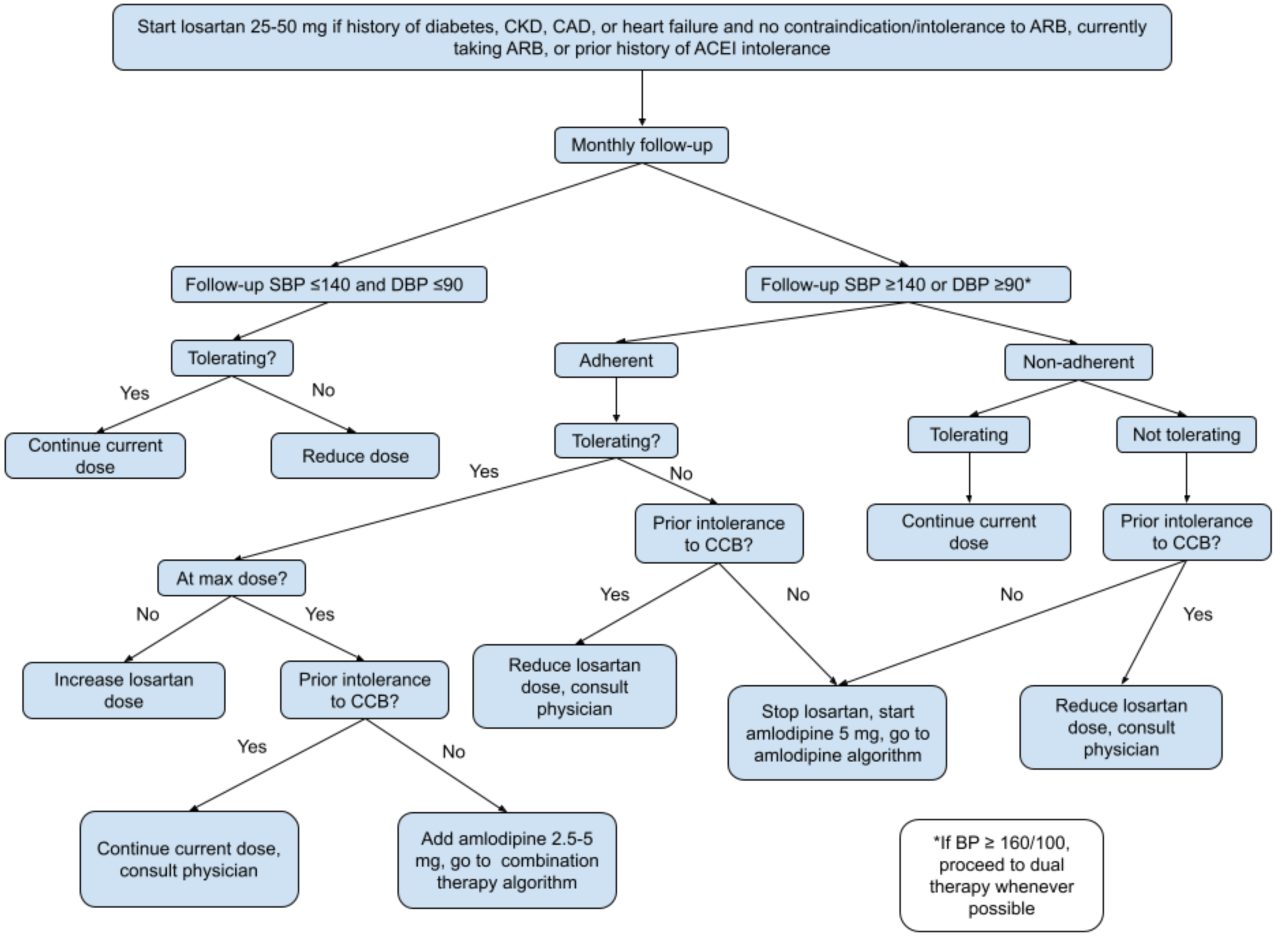

Dual antihypertensive therapy titration algorithm

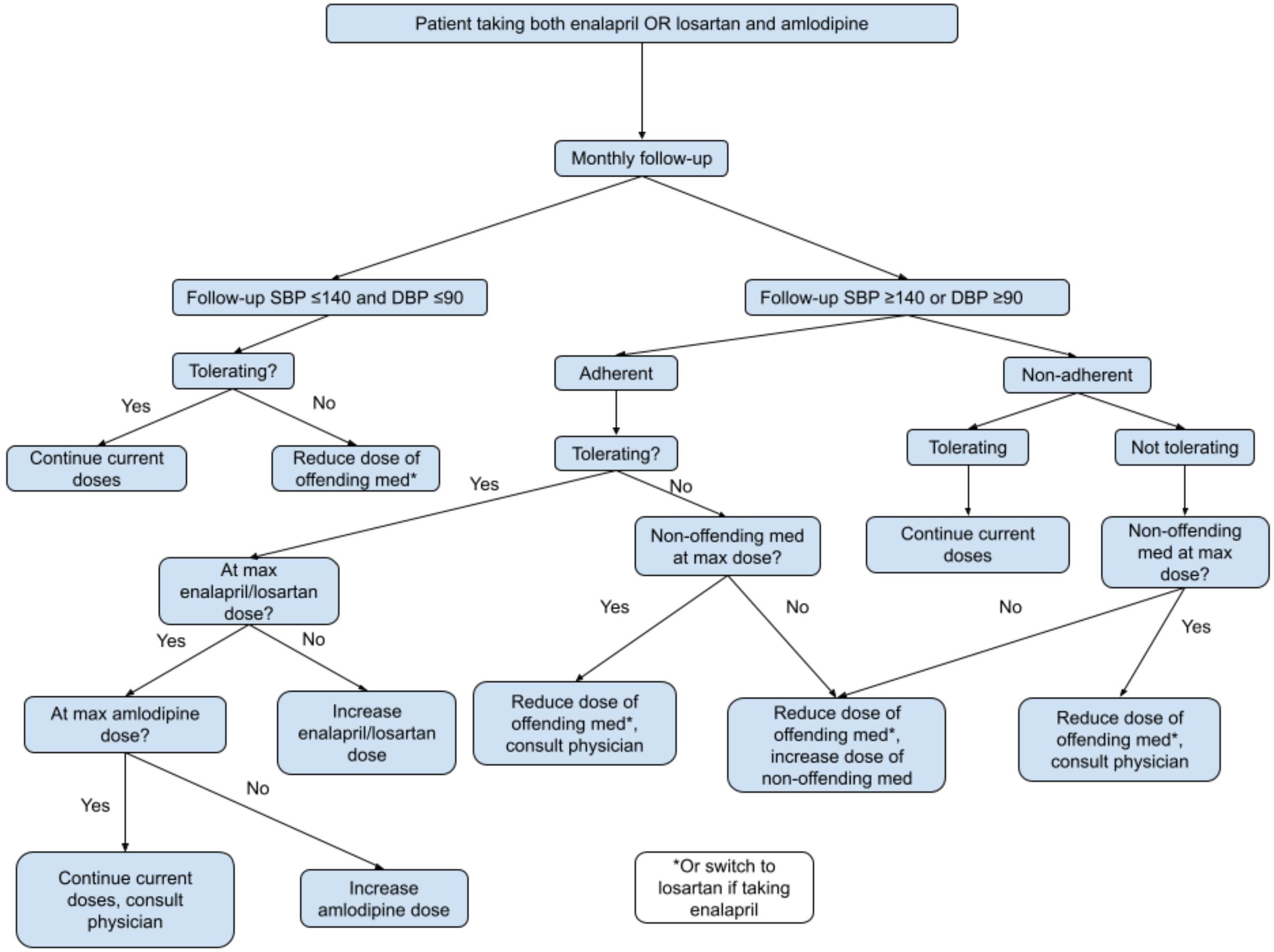

Appendix S2 - Automated criteria for determining medication contraindications

- Enalapril/ACEI
  - Woman of childbearing potential:
    - No history of hysterectomy or tubal ligation
    - Has not gone through menopause
    - < 50 years of age
  - History of angioedema with ACEI or ARB
  - History of cough with ACEI
  - History of severe allergy to ACEI (e.g. anaphylaxis)
- Losartan/ARB
  - Woman of childbearing potential
  - History of angioedema with ARB
  - History of severe allergy to ARB
- Amlodipine
  - History of severe allergy to amlodipine
  - History of gingival hyperplasia
- Atorvastatin
  - Woman of childbearing potential
  - History of severe myopathy/rhabdomyolysis with statin
  - History of hepatitis secondary to statin
  - Current hepatitis/active liver disease
  - History of severe allergic reaction to statin
- Aspirin
  - History of severe aspirin allergy
  - History of hemorrhagic stroke

Appendix S3 - CONSORT 2010 pilot and feasibility studies extension reporting checklist

CONSORT 2010 checklist of information to include when reporting a pilot or feasibility trial*

Section/Topic	Item No	Checklist item	Reported on page No
**Title and abstract**			
	1a	Identification as a pilot or feasibility randomised trial in the title	1

	1b	Structured summary of pilot trial design, methods, results, and conclusions (for specific guidance see CONSORT abstract extension for pilot trials)	2
**Introduction**			
Background and objectives	2a	Scientific background and explanation of rationale for future definitive trial, and reasons for randomised pilot trial	3
	2b	Specific objectives or research questions for pilot trial	4
**Methods**			
Trial design	3a	Description of pilot trial design (such as parallel, factorial) including allocation ratio	7
	3b	Important changes to methods after pilot trial commencement (such as eligibility criteria), with reasons	NA
Participants	4a	Eligibility criteria for participants	7
	4b	Settings and locations where the data were collected	4
	4c	How participants were identified and consented	7
Interventions	5	The interventions for each group with sufficient details to allow replication, including how and when they were actually administered	8
Outcomes	6a	Completely defined prespecified assessments or measurements to address each pilot trial objective specified in 2b, including how and when they were assessed	9
	6b	Any changes to pilot trial assessments or measurements after the pilot trial commenced, with reasons	NA
	6c	If applicable, prespecified criteria used to judge whether, or how, to proceed with future definitive trial	9, 10
Sample size	7a	Rationale for numbers in the pilot trial	7
	7b	When applicable, explanation of any interim analyses and stopping guidelines	NA
Randomisation:			
Sequence generation	8a	Method used to generate the random allocation sequence	NA
	8b	Type of randomisation(s); details of any restriction (such as blocking and block size)	NA
Allocation concealme nt mechanis m	9	Mechanism used to implement the random allocation sequence (such as sequentially numbered containers), describing any steps taken to conceal the sequence until interventions were assigned	NA
Implementation	10	Who generated the random allocation sequence, who enrolled participants, and who assigned participants to interventions	NA
Blinding	11a	If done, who was blinded after assignment to interventions (for example, participants, care providers, those assessing outcomes) and how	NA
	11b	If relevant, description of the similarity of interventions	NA
Statistical methods	12	Methods used to address each pilot trial objective whether qualitative or quantitative	11
**Results**			
Participant flow (a diagram is strongly recommended)	13a	For each group, the numbers of participants who were approached and/or assessed for eligibility, randomly assigned, received intended treatment, and were assessed for each objective	NA
	13b	For each group, losses and exclusions after randomisation, together with reasons	15

Recruitment	14a	Dates defining the periods of recruitment and follow-up	11
	14b	Why the pilot trial ended or was stopped	NA
Baseline data	15	A table showing baseline demographic and clinical characteristics for each group	12
Numbers analysed	16	For each objective, number of participants (denominator) included in each analysis. If relevant, these numbers should be by randomised group	13
Outcomes and estimation	17	For each objective, results including expressions of uncertainty (such as 95% confidence interval) for any estimates. If relevant, these results should be by randomised group	13
Ancillary analyses	18	Results of any other analyses performed that could be used to inform the future definitive trial	15, 17
Harms	19	All important harms or unintended effects in each group (for specific guidance see CONSORT for harms)	18
	19a	If relevant, other important unintended consequences	18
**Discussion**			
Limitations	20	Pilot trial limitations, addressing sources of potential bias and remaining uncertainty about feasibility	26
Generalisability	21	Generalisability (applicability) of pilot trial methods and findings to future definitive trial and other studies	26
Interpretation	22	Interpretation consistent with pilot trial objectives and findings, balancing potential benefits and harms, and considering other relevant evidence	21
	22a	Implications for progression from pilot to future definitive trial, including any proposed amendments	26
**Other information**			
Registration	23	Registration number for pilot trial and name of trial registry	27
Protocol	24	Where the pilot trial protocol can be accessed, if available	7
Funding	25	Sources of funding and other support (such as supply of drugs), role of funders	28
	26	Ethical approval or approval by research review committee, confirmed with reference number	27

Citation: Eldridge SM, Chan CL, Campbell MJ, Bond CM, Hopewell S, Thabane L, et al. CONSORT 2010 statement: extension to randomised pilot and feasibility trials. BMJ. 2016;355. This is an Open Access article distributed in accordance with the terms of the Creative Commons Attribution (CC BY 3.0) license (http://creativecommons.org/licenses/by/3.0/), which permits others to distribute, remix, adapt and build upon this work, for commercial use, provided the original work is properly cited.

*We strongly recommend reading this statement in conjunction with the CONSORT 2010, extension to randomised pilot and feasibility trials, Explanation and Elaboration for important clarifications on all the items. If relevant, we also recommend reading CONSORT extensions for cluster randomised trials, non-inferiority and equivalence trials, non-pharmacological treatments, herbal interventions, and pragmatic trials. Additional extensions are forthcoming: for those and for up-to-date references relevant to this checklist, see www.consort-statement.org.

Appendix S4 - Standardized protocol for blood pressure measurement

1. Prior to measurement, patient should have an empty bladder and not have smoked, drunk coffee, or exercised for 30 minutes

2. Patient sitting and relaxed for at least 5 minutes prior to the measurement
3. Cuff is placed on bare skin on the middle of the left arm and maintained at the level of the heart during the measurement.
4. During and between measurements: neither patient nor evaluator talks; back is supported; feet are maintained on the ground; legs are not crossed
5. If the initial BP is ≥130/85 mmHg, two more measurements are taken at 1 minute intervals and the arithmetic mean of these two measurements is used.

Appendix S5 - Semi-structured interview guide

1. How satisfied are you with your current treatment for hypertension?
2. How has the hypertension program helped you to control your blood pressure?
3. How does your treatment in the hypertension program compare to treatment you received previously?
4. What about the hypertension program are you most satisfied with?
5. What could we do to improve the hypertension program and better help you to control your blood pressure?

Table S1 - Blood pressure control status using combined systolic and diastolic pressure

	Control at 6 months		
Baseline Control	No	Yes	Total
No	20.0% (6)	20.0% (6)	40.0% (12)

Yes	10.0% (3)	50.0% (15)	60.0% (20)
Total	30.0 % (9)	70.0% (21)	100.0% (30)

Table S2 - PACIC survey results

Commented [A1]: Please add to supplement

Characteristic	Baseline N = 30*	6 months N = 30*	Difference†	95% CI†	p-value†
Asked for my ideas when we made a treatment plan	5.0 (4.0, 5.0)	5.0 (4.0, 5.0)	0.50	-1.0, 1.5	0.3
Asked to talk about any problems with my medicines or their effects	5.0 (4.0, 5.0)	5.0 (5.0, 5.0)	1.0	3.3×10^{-5}, 2.0	0.039
Given a written list of things I should do to improve my health	5.0 (3.0, 5.0)	5.0 (5.0, 5.0)	2.0	0.50, 3.0	0.007
Satisfied that my care was well organized	5.0 (5.0, 5.0)	5.0 (5.0, 5.0)	-3.4×10^{-5}	-1.0, 0.50	0.82
Shown how what I did to take care of my illness influenced my condition	5.0 (4.0, 5.0)	5.0 (5.0, 5.0)	1.5	4.6×10^{-5}, 2.5	0.025

Helped to set specific goals to improve my eating or exercise	5.0 (3.0, 5.0)	5.0 (5.0, 5.0)	1.5	0.50, 2.5	0.005
Given a copy of my treatment plan	5.0 (3.0, 5.0)	5.0 (5.0, 5.0)	1.0	-1.9×10^{-5}, 2.5	0.11
Asked questions, either directly or on a survey, about my health habits	4.1 (1.2)	4.6 (0.8)	0.53	-0.069, 1.1	0.081
Asked what I would like to discuss about my illness at that visit	5.0 (3.0, 5.0)	5.0 (5.0, 5.0)	1.5	0.50, 2.5	0.015
Told how important the things I do to take care of my illness (e.g. exercise) were for my health	5.0 (4.0, 5.0)	5.0 (5.0, 5.0)	1.0	-4.2×10^{-5}, 2.0	0.052
Given a book or monitoring log in which to record the progress I am making	5.0 (3.0, 5.0)	5.0 (5.0, 5.0)	1.5	4.4×10^{-5}, 2.5	0.029
Average PACIC item score	4.5 (3.9, 5.0)	5.0 (4.6, 5.0)	0.50	0.14, 1.0	0.011

* Median (Q1, Q3); Mean (SD)

† Wilcoxon signed rank test with continuity correction (median difference reported); Paired t-test (mean difference reported)

Table S3 - Physician consults by reason for consult

Reason for consult	Number of consults (% of total consults) *
Scenario outside of antihypertensive titration protocol	11 (42.3)
CHW concern about patient	10 (38.5)
Chest pain	5 (19.2)
Heart failure symptoms	1 (3.8)

* Total N = 26. Percentages add up to more than 100% as some consults were for more than one reason

Table S4 - H-SCALE results

Characteristic	Baseline N = 30*	6 months N = 30*	Difference†	95% CI†	p-value†
Adherence Score	20.0 (15.0, 21.0)	17.0 (14.0, 21.0)	- 1.5	-4.5, 1.0	0.21
Diet Score	25.7 (11.0)	24.4 (7.9)	-1.3	-5.3, 2.6	0.5
Physical Activity Score	2.7 (2.5)	3.6 (3.0)	0.90	-0.22, 2.0	0.11

Resistance Activity Score	0.0 (0.0, 0.0)	0.0 (0.0, 0.0)	-0.15	-4.0, 3.5	0.85
Smoking Score	0.0 (0.0, 0.0)	0.0 (0.0, 0.0)	-1.0	-1, 1	0.089
Weight Control Score	36.6 (5.8)	37.5 (3.3)	0.93	-1.9, 3.8	0.5

Abbreviation: CI = Confidence Interval

* Median (Q1, Q3); Mean (SD)

† Wilcoxon signed rank test with continuity correction (median difference reported); Paired t-test (mean difference reported)

Table S5 - Laboratory results

**Characteristic**	**Baseline** N = 30*	**6 Months** N = 30*	**Difference†**	**95% CI†**	**p-value†**
Creatinine (mg/dL)	0.93 (0.38)	1.03 (0.44)	0.10	0.04, 0.15	0.001
eGFR (mL/min/1.73 m^2)	102 (28)	94 (26)	-7.6	-13, -1.8	0.011
Potassium (mEq/L)	4.50 (0.42)	4.47 (0.39)	-0.03	-0.19, 0.12	0.7

Total cholesterol (mg/dL)	177 (35)	170 (34)	-6.9	-17, 2.9	0.2
HDL cholesterol (md/dL)	52 (16)	54 (12)	2.7	-2.9, 8.3	0.3
LDL cholesterol (mg/dL)	76 (26)	80 (30)	3.7	-7.7, 15	0.5
Triglycerides (mg/dL)	200 (164, 262)	132 (120, 219)	-60.0	-93.5, -34.5	<0.001
Glucose (mg/dL)	108 (85, 125)	90 (79, 123)	-11.5	-19.0, -0.5	0.041
ALT (U/L)	31 (15)	25 (15)	-6.6	-11, -2.4	0.003

Abbreviation: CI = Confidence Interval

* Mean (SD); Median (Q1, Q3)

† Paired t-test (mean difference reported); Wilcoxon signed rank test with continuity correction (median difference reported)

Table S6 - Changes in weight and BMI

**Characteristic**	**Baseline** N = 30*	**6 months** N = 30*	**Difference†**	**95% CI†**	**p-value†**
BMI (kg/m^2)	29.2 (4.7)	29.1 (4.7)	-0.15	-0.56, 0.26	0.5

Weight (lbs)	134.9 (26.5)	134.1 (26.1)	-0.85	-2.7, 1.0	0.4

Abbreviation: CI = Confidence Interval

* Mean (SD)

† Paired t-test

Table S7 - Medication adherence at baseline and six months

	Adherence at 6 months†		
Baseline Adherence*	Yes	No	Total
Yes	96% (27)	4% (1)	100% (28)
No	0% (0)	0% (0)	0% (0)
Total	96% (27)	4% (1)	100% (28) ‡

* Using self-report, as PCAR was not available at baseline

† Using PCAR if available, or self-report if valid PCAR not available

‡ This analysis includes the 28 participants who were taking medications at baseline and at 6 months

Table S8 - Medication adherence calculated with PCAR

	Adherence at 6 months (PCAR)		
Adherence at 1 month (PCAR)	Yes	No	Total

Yes	72% (10)	21% (3)	93% (13)
No	7% (1)	0% (0)	7% (1)
Total	79% (11)	21% (3)	100% (14) *

* This analysis includes the 14 participants for whom a valid PCAR could be calculated at 1 month and 6 months
